# Tabetic Arthropathy as an Uncommon Cause of Destructive Joint Disease: A Case Report

**DOI:** 10.7759/cureus.104897

**Published:** 2026-03-09

**Authors:** Siham Rachidi, Saadia Ait Malek, Erraoui Mariam, Imad Ghozlani

**Affiliations:** 1 Rheumatology, University Hospital of Mohammed VI, Agadir, MAR; 2 Rheumatology, Cartilage and Bone (CARBONE) Research Team, Research Laboratory of Innovation in Health Sciences (LARISS) Faculty of Medicine and Pharmacy of Agadir, Ibn Zohr University, Agadir, MAR; 3 Rheumatology, Oued Eddahab Military Hospital, Agadir, MAR

**Keywords:** knee joints, late neurosyphilis, penicillin g, serology testing, tabetic arthropathy

## Abstract

Tabetic arthropathy is a destructive neurogenic joint disease that has become rare due to the early treatment of syphilis with penicillin G. It results from neurosyphilis caused by *Treponema pallidum*. The diagnosis is suspected in patients presenting with severe joint destruction disproportionate to the absence or minimal intensity of pain and is confirmed by positive syphilitic serological tests.

We report the case of a 62-year-old patient with a history of high-risk sexual behavior and a remote episode of cervical lymphadenopathy treated 40 years ago. He presented with chronic painless monoarthritis of the right knee, without fever, associated with elevated inflammatory markers. Knee radiography revealed destruction of the medial tibial condyle, while magnetic resonance imaging (MRI) demonstrated osteolytic lesions of the medial tibial plateau with bone fragment detachment. The diagnosis of tabetic arthropathy was confirmed by positive syphilis serology. The clinical course was favorable following appropriate treatment, with marked clinical and biological improvement.

Although exceptional, tabetic arthropathy should be considered in cases of painless destructive arthropathy. Treatment is primarily based on intravenous penicillin G, with third-generation cephalosporins as an alternative when penicillin is unavailable.

## Introduction

Tabetic arthropathy is a destructive neurogenic joint disease that occurs as a late complication of neurosyphilis, accounting for approximately 4%-10% of tabes dorsalis cases. Although it has become exceptional in current clinical practice due to the early management of syphilis with penicillin G, it remains a potentially severe and disabling condition [1]. The causative pathogen is *Treponema pallidum*.

Clinically, the diagnosis is suggested by a marked discrepancy between the severity of joint destruction and the absence or minimal intensity of pain, reflecting the underlying sensory neuropathy. Biological confirmation relies on positive syphilitic serological tests in the blood, while imaging plays a key role in assessing the extent of articular damage [2].

Despite its rarity, the global resurgence of syphilis over recent decades raises renewed concern for late and atypical manifestations. In modern rheumatology practice, painless destructive arthropathy may be misdiagnosed as rapidly progressive osteoarthritis or inflammatory arthritis, leading to diagnostic delay. This case is reported to highlight tabetic arthropathy as an important differential diagnosis of rapidly progressive, painless joint destruction and to emphasize the need for maintaining clinical awareness of neurosyphilis despite its rarity.

## Case presentation

We report the case of a 62-year-old married man with a history of unprotected sexual intercourse and a remote episode of chronic cervical lymphadenopathy treated approximately 40 years earlier. He presented to our department in January 2026 with chronic monoarthritis of the right knee, evolving over nine months, associated with partial functional limitation. The clinical course was insidious, with no fever and preserved general condition.

**Figure 1:**
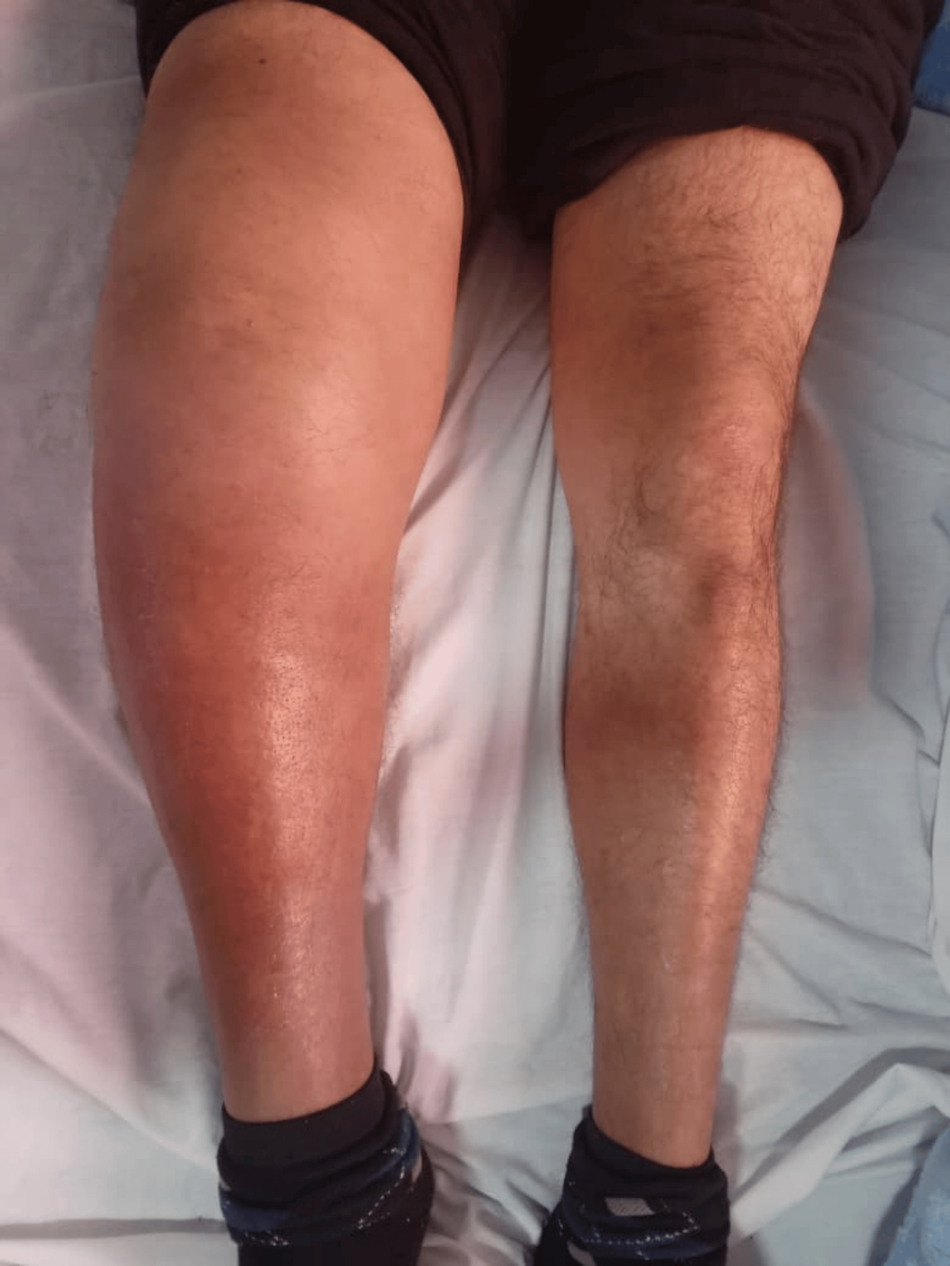
Monoarthritis of the right knee with inflammatory signs and marked periarticular soft tissue infiltration

On clinical examination, the patient was conscious and fully oriented to time and place. Vital signs were stable, with an axillary temperature of 37°C, blood pressure of 130/70 mmHg, and a regular heart rate of 80 beats per minute. Musculoskeletal examination revealed a deformed, swollen, and warm right knee, which was remarkably painless, with marked periarticular soft tissue infiltration (Figure 1). The contralateral knee was normal. Neurological examination demonstrated hypoesthesia in the lower limbs, with associated areflexia, gait ataxia, and hypotonia.

Laboratory investigations revealed a hemoglobin level of 12.4 g/dL, a platelet count of 429,000/mm³, and a normal leukocyte count of 7,520/mm³. Renal function was preserved, with a serum creatinine level of 7.9 mg/L. Inflammatory markers were significantly elevated, including a C-reactive protein level of 28.46 mg/L and an erythrocyte sedimentation rate of 88 mm in the first hour. Serum uric acid level was within the normal range (57 mg/L). Laboratory findings are summarized in Table 1.

**Table 1:** Laboratory test findings with reference ranges AST: Aspartate aminotransferase; ALT: Alanine aminotransferase; CRP: C-reactive protein; ESR: Erythrocyte sedimentation rate.

Parameters	Patient value	Reference ranges
White blood cell count (cells/mm³)	7,520	4,000–10,000
Hemoglobin (g/dL)	12.4	13–17
Platelet count (cells/mm³)	429,000	150,000–450,000
Serum urea (g/L)	0.30	0.15–0.45
Serum creatinine (mg/L)	7.36	7–12.5
AST (IU/L)	23	<42
ALT (IU/L)	12	<41
CRP (mg/L)	28.46	<5
ESR (mm/h)	88	2–10

**Figure 2:**
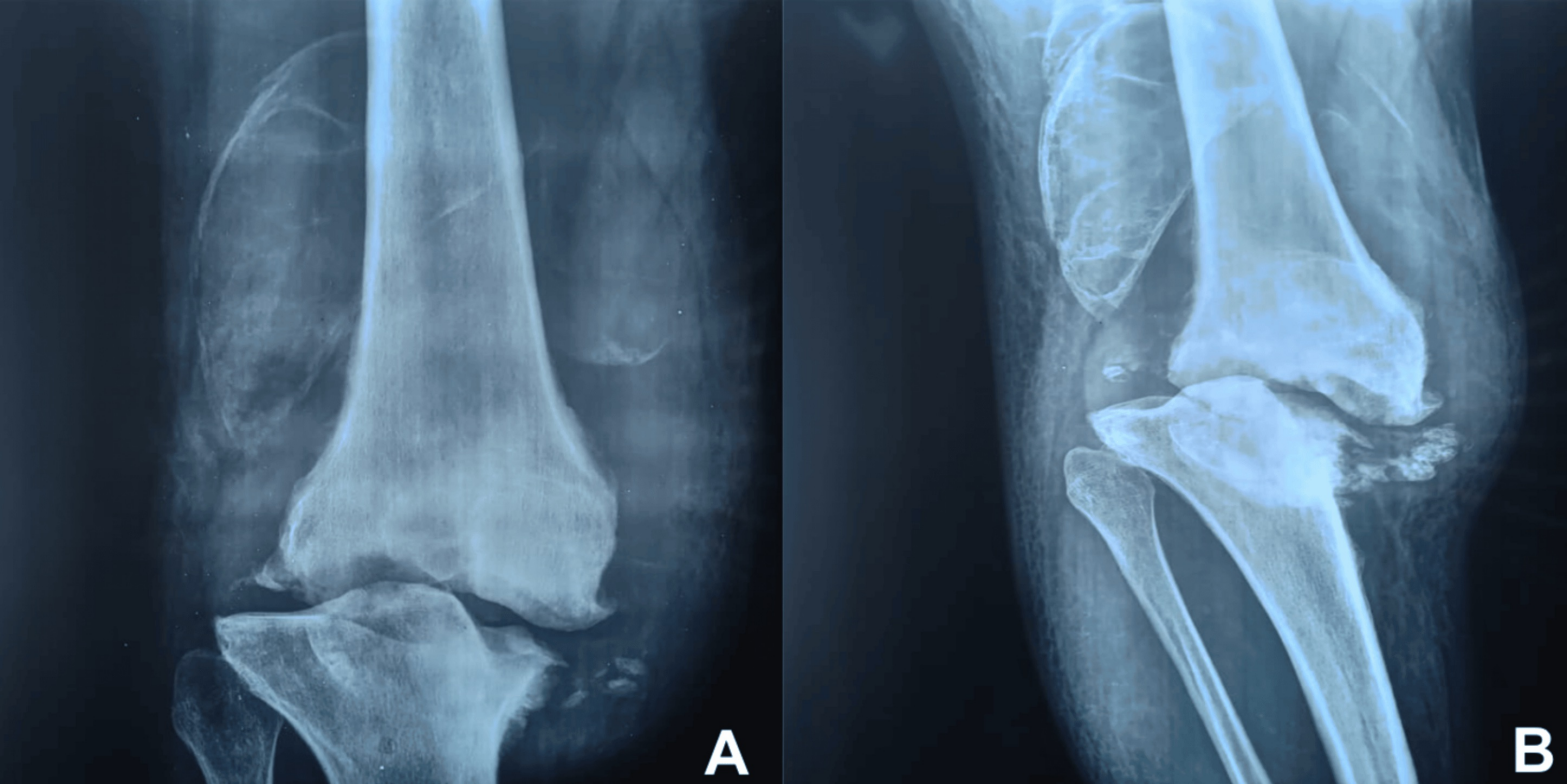
Radiographic findings of the right knee: (A) anteroposterior and (B) lateral radiographs Anteroposterior and lateral radiographs of the right knee demonstrating severe destruction of the medial femorotibial compartment, with osteolysis of the medial tibial plateau and associated osteophytic bone formations.

Synovial fluid analysis revealed a hemorrhagic fluid that was sterile on bacteriological culture and devoid of crystals. Standard X-ray of the right knee (Figure 2) demonstrated severe destruction of the medial femorotibial compartment, with osteolysis of the medial margin of the medial tibial plateau, associated with osteophytic formations and intra-articular fragments.

**Figure 3:**
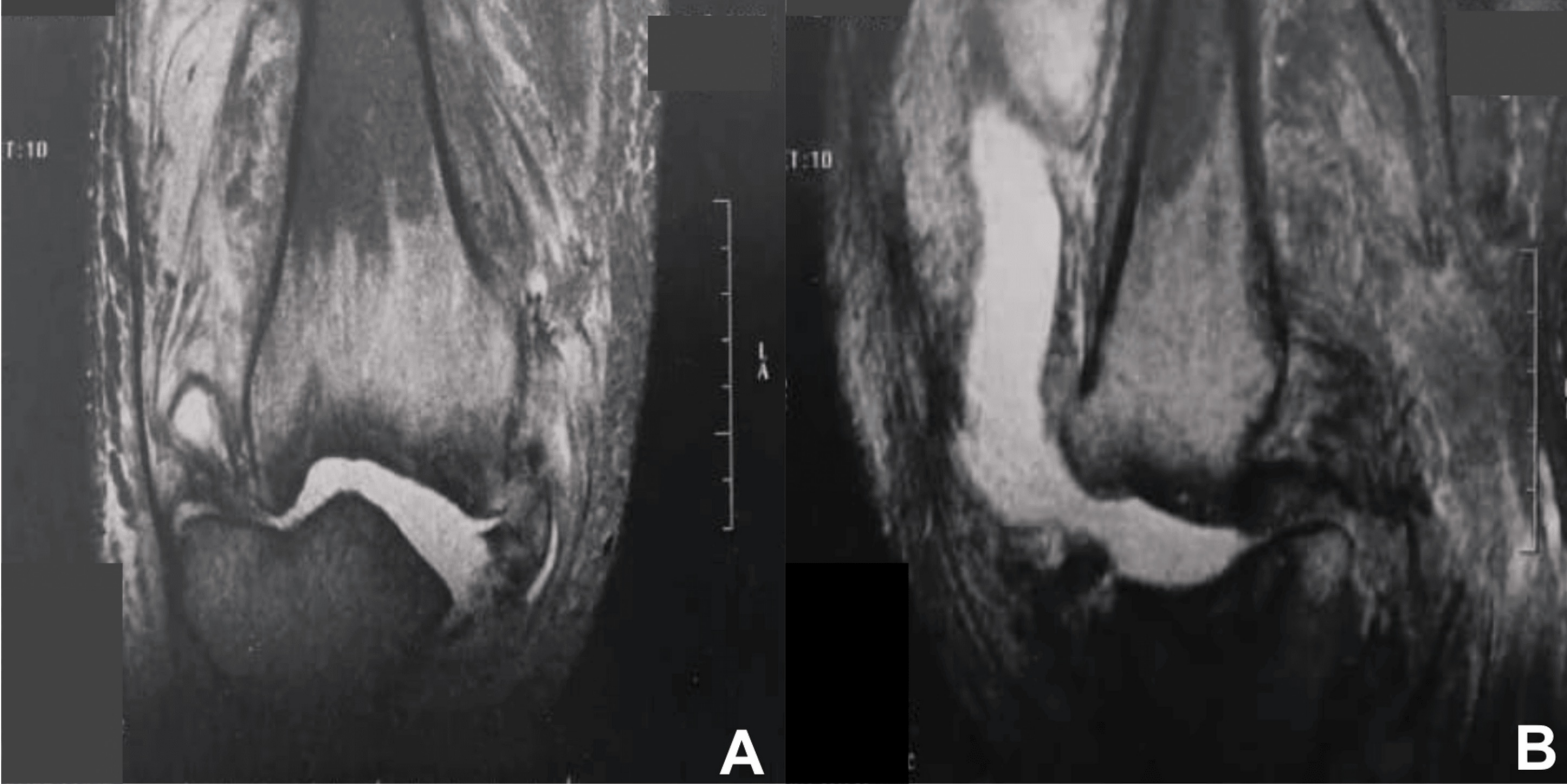
MRI of the right knee: (A) sagittal T2-weighted sequence and (B) sagittal T2-weighted STIR sequence MRI sequences of the right knee showing extensive destruction of the medial femoral condyle with osteolysis of the medial tibial plateau associated with periarticular bone remodeling. STIR: Short tau inversion recovery.

Musculoskeletal ultrasound revealed a large joint effusion with synovial hypertrophy, suggestive of synovial hydrarthrosis. MRI of the knee (Figure 3) showed a massive intra-articular fluid effusion, diffuse infiltration of the subcutaneous soft tissues, and peripheral synovial enhancement following gadolinium administration. Extensive osteolytic lesions were noted involving both tibial plateaus, the femoral condyles, and, partially, the patella, associated with periarticular osteophytic remodeling. MRI also demonstrated complete ruptures of the anterior and posterior cruciate ligaments, distension and elongation of the collateral ligaments, meniscal dislocation with fracture of the posterior horns of the right knee, and subluxation of both the femorotibial and femoropatellar joints.

**Figure 4:**
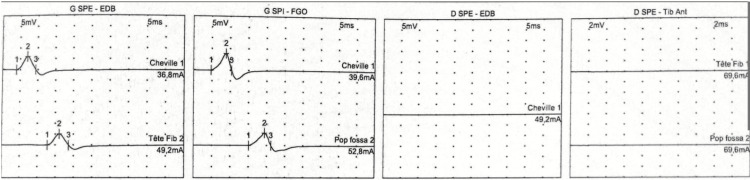
Electroneuromyography showing severe motor involvement of the right common peroneal nerve SPE: Common peroneal nerve; EDB: Extensor digitorum brevis muscle; SPI: Posterior tibial nerve; FGO: Flexor digitorum muscle of the foot.

Electroneuromyography (Figure 4) revealed a marked reduction in the motor amplitude of the right common peroneal nerve. Interpretation was limited by the presence of lower limb edema.

Serological testingfor syphilis was positive in the blood, with a positive *Treponema pallidum* hemagglutination assay (TPHA) and a negative Venereal Disease Research Laboratory (VDRL) test, consistent with a past syphilitic infection (Table 2). Based on the clinical, radiological, and serological findings, a diagnosis of hypertrophic tabetic arthropathy was established. Although cerebrospinal fluid (CSF) analysis is recommended to confirm neurosyphilis, it was not performed due to the patient's refusal of a lumbar puncture. Owing to the unavailability of penicillin G, the patient was treated with intravenous third-generation cephalosporins at a dose of 2 g/day for 15 days. Following antibiotic therapy, the patient showed marked clinical improvement, with a significant reduction of joint swelling, stabilization of symptoms, and normalization of inflammatory markers, as reflected by a decrease in C-reactive protein levels to 5 mg/L. Given the advanced structural damage, orthopedic management was discussed, and conservative treatment with joint offloading was maintained.

**Table 2:** Syphilis serological test results TPHA: *Treponema pallidum* hemagglutination assay; VDRL: Venereal Disease Research Laboratory test.

Test	Patient result	Reference range	Interpretation
TPHA	Positive	Negative	Consistent with previous exposure to *Treponema pallidum*
VDRL	Negative	Negative	No evidence of active syphilis

## Discussion

Syphilis is a sexually transmitted infection whose incidence has been increasing worldwide over the past two decades. It is caused by *T. pallidum*, a thin, non-cultivable Gram-negative spirochete with an exclusively human reservoir. Diagnosis relies on the combined use of non-treponemal tests, such as the VDRL, and treponemal tests, including the TPHA. The clinical presentation of syphilis is highly polymorphic, which explains the frequent diagnostic delays, particularly in the late stages of the disease.

Tertiary syphilis represents the most severe form of infection and may manifest as cardiovascular involvement (aortic aneurysm or aortic regurgitation), neurosyphilis, or auditory and ocular complications. Neurosyphilis can occur at different stages of the disease. Meningeal involvement is relatively frequent but remains clinically silent in most cases, becoming symptomatic in only 5%-10% of patients. Late neurosyphilis includes tabes dorsalis, gummatous lesions, general paresis, and cognitive impairment resembling a frontal dementia syndrome [3].

Tabetic arthropathy is a rare but severe complication of neurosyphilis. It predominantly affects men and typically occurs late, between the fifth and sixth decades of life, after a prolonged asymptomatic period that may exceed 15 years following primary infection [1]. It accounts for approximately 4%-10% of complications of tabes dorsalis. This condition corresponds to a destructive neurogenic arthropathy resulting from the loss of pain perception and proprioceptive sensation, leading to repetitive microtrauma and progressive joint destruction [4]. First described by Charcot in 1868 and later further characterized by Kroenig and Abadie, tabetic arthropathy remains a historical yet still relevant clinical entity [2].

The pathophysiology of tabetic arthropathy remains incompletely understood. Two main hypotheses have been proposed. The trophic theory implicates autonomic nervous system dysfunction, particularly sympathetic involvement, resulting in vasomotor disturbances, impaired joint nutrition, and increased osteoclastic bone resorption. The mechanical theory attributes joint destruction to anesthesia and proprioceptive loss, which abolish protective mechanisms and expose the joint to repeated microtrauma, facilitated by ataxia and ligamentous hyperlaxity [5].

Clinically, tabetic arthropathy is characterized by a striking discrepancy between the severity of joint destruction and the minimal or absent pain. All joints may be affected; however, the lower limbs are most commonly involved, particularly the knee, followed by the hip. Early forms may mimic common osteoarthritis, contributing to diagnostic delay [1]. The disease is most often monoarticular or pauciarticular, although polyarticular forms have been reported. Spinal involvement is relatively frequent, occurring in approximately 20% of cases, mainly affecting the lumbar spine, while small joints and sacroiliac joints are rarely involved [6].

Radiographic findings evolve progressively, ranging from early degenerative changes, such as osteosclerosis, subchondral cysts, and osteophyte formation, to advanced forms characterized by massive intra- and extra-articular bone destruction, fragmentation, large geodes, subchondral erosions, intra-articular loose bodies, and occasionally osteonecrosis. Magnetic resonance imaging (MRI) allows detailed assessment of bone, ligamentous, and soft tissue involvement, thereby facilitating diagnostic confirmation and disease staging [7].

From a serological standpoint, the VDRL test, although nonspecific, is a semiquantitative marker of disease activity. It becomes positive approximately two weeks after chancre onset and often becomes negative or weakly positive in late stages. In contrast, TPHA is more sensitive and specific, becoming positive around the fourth week and remaining positive for life [8]. Other treponemal tests, such as the fluorescent treponemal antibody absorption (FTA-ABS), become positive early but do not distinguish between active and past infection [3]. Cerebrospinal fluid analysis may support the diagnosis of neurosyphilis, showing lymphocytic pleocytosis, elevated protein levels, normal glucose concentration, and positive serology, although it may be normal in up to 20% of patients with established disease [2].

Classically, the diagnosis of tabetic arthropathy is suggested by the combination of painless joint deformation, rapid structural deterioration, and radiological osteolysis, with or without associated neurological symptoms. Differential diagnoses include pseudo-neurogenic calcium pyrophosphate deposition disease, rapidly progressive osteoarthritis, and other neurogenic arthropathies, particularly syringomyelia.

The treatment of choice relies on parenteral penicillin G administered for 10-15 days. Therapeutic alternatives are limited and less well validated [9]. Orthopedic management is often disappointing, particularly in advanced stages, where surgical procedures such as arthrodesis may be reserved for cases of severe instability. In early stages, conservative measures including rest, immobilization, and joint offloading may be sufficient. Prognosis largely depends on the stage at diagnosis and the prompt initiation of appropriate therapy [10].

## Conclusions

Tabetic arthropathy is an exceptional but severe complication of neurosyphilis that is frequently overlooked. It should be considered in any patient presenting with progressive, painless, destructive arthropathy, particularly in the presence of risk factors for past syphilitic infection. Early diagnosis is crucial, as timely treatment with penicillin G remains the only effective means of preventing irreversible neurological and articular damage, whose management remains challenging and often unsatisfactory.
